# Pre-sleep protein supplementation does not improve recovery from load carriage in British Army recruits (part 2)

**DOI:** 10.3389/fnut.2023.1264042

**Published:** 2023-11-30

**Authors:** Shaun Chapman, Justin Roberts, Andrew J. Roberts, Henry Ogden, Rachel Izard, Lee Smith, Havovi Chichger, Lauren Struszczak, Alex J. Rawcliffe

**Affiliations:** ^1^Army Recruit Health and Performance Research, HQ Army Recruiting and Initial Training Command, Medical Branch, UK Ministry of Defence, Upavon, United Kingdom; ^2^Cambridge Centre for Sport and Exercise Sciences, School of Psychology and Sport Science, Anglia Ruskin University, Cambridge, United Kingdom; ^3^Defence Science and Technology, UK Ministry of Defence, Salisbury, United Kingdom; ^4^Centre for Health, Performance and Wellbeing, Anglia Ruskin University, Cambridge, United Kingdom; ^5^Biomedical Science Research Group, School of Life Science, Anglia Ruskin University, Cambridge, United Kingdom; ^6^Public Health and Sports Sciences, University of Exeter, Exeter, United Kingdom; ^7^Faculty of Science and Engineering, Anglia Ruskin University, Cambridge, United Kingdom

**Keywords:** nutrition, muscle damage, military, repair, amino acids

## Abstract

British Army basic training (BT) is physically demanding with new recruits completing multiple bouts of physical activity each day with limited recovery. Load carriage is one of the most physically demanding BT activities and has been shown to induce acute exercise-induced muscle damage (EIMD) and impair muscle function. Protein supplementation can accelerate muscle recovery by attenuating EIMD and muscle function loss. This study investigated the impact of an additional daily bolus of protein prior to sleep throughout training on acute muscle recovery following a load carriage test in British Army recruits. Ninety nine men and 23 women (mean ± SD: age: 21.3 ± 3.5 yrs., height: 174.8 ± 8.4 cm, body mass 75.4 ± 12.2 kg) were randomized to dietary control (CON), carbohydrate placebo (PLA), moderate (20 g; MOD) or high (60 g; HIGH) protein supplementation. Muscle function (maximal jump height), perceived muscle soreness and urinary markers of muscle damage were assessed before (PRE), immediately post (POST), 24-h post (24 h-POST) and 40-h post (40 h-POST) a load carriage test. There was no impact of supplementation on muscle function at POST (*p* = 0.752) or 40 h-POST (*p* = 0.989) load carriage but jump height was greater in PLA compared to HIGH at 24 h-POST (*p* = 0.037). There was no impact of protein supplementation on muscle soreness POST (*p* = 0.605), 24 h-POST (*p* = 0.182) or 40 h-POST (*p* = 0.333). All groups had increased concentrations of urinary myoglobin and 3-methylhistidine, but there was no statistical difference between groups at any timepoint (*p* > 0.05). We conclude that pre-sleep protein supplementation does not accelerate acute muscle recovery following load carriage in British Army recruits during basic training. The data suggests that consuming additional energy in the form of CHO or protein was beneficial at attenuating EIMD, although it is acknowledged there were no statistical differences between groups. Although EIMD did occur as indicated by elevated urinary muscle damage markers, it is likely that the load carriage test was not arduous enough to reduce muscle function, limiting the impact of protein supplementation. Practically, protein supplementation above protein intakes of 1.2 g⸱kg^−1^⸱day^−1^ following load carriage over similar distances (4 km) and carrying similar loads (15–20 kg) does not appear to be warranted.

## 1 Introduction

British Army Basic Training (BT) is a physically demanding 14-week course with new recruits completing multiple bouts of physical activity with limited recovery during a 24-h period (1–3). New recruits fail to meet the recommended energy, carbohydrate, and protein intakes during BT (2, 4) with limited energy and negligible protein intakes between meals, particularly in the evening (3). In the first article of this dual submission, pre-sleep protein supplementation did not impact physical performance, body composition or chronic recovery during BT. This second article investigates the impact of pre-sleep protein supplementation on acute muscle recovery following a standardized British Army load carriage test during BT. The ability to carry load over long distances is fundamental to many roles within the military (5) and is a key activity during British Army BT. Load carriage is considered one of the most physically demanding tasks during military training and has been shown to impair skeletal muscle function (5–7). A reduction in muscle function following load carriage during military recruit training can impair acute physical performance (6). Maximal voluntary contraction (MVC) of the knee extensors was reduced by 12 ± 9% and 9 ± 13% in male and female recruits within 30 min following load carriage over ~10 km carrying 15–20 kg (5). It has also been reported that vertical jump height was significantly reduced by 5 ± 11% and 5 ± 6% in men and women, respectively (5). Such reductions in muscle function could impair training and increase injury risk in the days following load carriage (8). New recruits complete multiple bouts of physical activity during a 24-h period with limited recovery time (3), including following arduous activities such as load carriage. Therefore, strategies which accelerate muscle recovery are likely to be beneficial at maintaining military performance and reducing injury risk by limiting muscle damage and muscle function loss (8, 9). These are key aims for the military, particularly given the financial and operational cost associated with lost training days and medical discharges due to injury (10–12).

To date, protein supplementation has been investigated during military training in the United States (9, 13, 14) with scarce data in British Army recruits. These studies have also mostly focused on chronic training recovery and performance outcomes, not an acute single arduous military training event. Increased availability of protein, particularly the essential amino acids, attenuates muscle protein breakdown via hyperinsulinemia (15) and can accelerate acute skeletal muscle recovery by attenuating EIMD (16–20). For instance, Howatson et al. (17) reported attenuated muscle soreness and creatine kinase concentrations as well as better preservation of MVC of the knee extensors at 24–48 h post-exercise with branched-chain amino acid supplementation compared to a placebo. Branched-chain amino acids (3.5 g leucine; 2.1 g isoleucine; 1.7 g valine) have also been shown to reduce muscle soreness at 48 h post-exercise following resistance training in non-weight trained men (21). Nevertheless, the applicability of these studies to military recruits following load carriage exercise should be interpreted with caution, and specific studies are warranted. These previous studies included resistance-type exercise only which is not representative of military recruit populations (17, 20, 21). Additionally, previous studies have also included untrained individuals (21). Typically, EIMD is greater in untrained individuals and/or in response to a new training stimulus (22). Furthermore, load carriage is not exclusively a form of resistance exercise and is considered a form of concurrent exercise (23) and therefore, the degree of EIMD may not be comparable during load carriage and resistance or endurance exercise. Nevertheless, load carriage has consistently been shown to reduce acute muscle function (5–8). As such, dietary interventions which can potentially accelerate recovery or minimize the loss in muscle function following load carriage could be beneficial at maintaining occupational performance in the days following load carriage.

A limited number of studies have examined the effects of protein supplementation on acute skeletal muscle recovery following load carriage and have reported equivocal results. Post-exercise protein supplementation (24 g) significantly reduced self-perceived muscle soreness by −7% compared to increases of +10% and + 16% in the placebo and control conditions, respectively, following a six-mile hike in Royal Marines (24). Conversely, Blacker et al. (6) reported no effect of post-exercise protein supplementation (72 g) on knee extensor MVC at 48 and 72-h compared to an isocaloric placebo [carbohydrate (CHO)]. Similarly, no statistical differences were found between protein (25 g) and isocaloric CHO conditions when serum concentrations of cortisol, C-reactive protein, creatine kinase or aldolase were measured following a load carriage exercise (25). The limited number of studies and the differences between methodologies and outcome measures make it difficult to determine whether protein supplementation improves muscle recovery, thus warranting further research. Previous research has observed British Army recruits consume negligible amounts of protein in the evening time (3). Consuming protein prior to sleep supports muscle protein synthesis during sleep (26) but the impact on functional muscle recovery in the days following is equivocal with some (27) but not all observing accelerated muscle recovery (28–30) over 12–60 h post-exercise. It is acknowledged that the total daily amount of protein is considered more important than timing for muscle recovery outcomes. As such, the results of this study may provide support to the implementation of nutritional supplementation in the evening period to increase total daily protein intake and accelerate muscle recovery to maintain occupational performance and reduce injury risk. The aim of this study was to establish the extent to which an additional daily intake of a moderate (20 g) and high (60 g) bolus of protein prior to sleep influences acute muscle recovery following a load carriage test in British Army recruits.

## 2 Materials and methods

### 2.1 Study design and ethical approval

This randomized controlled trial assigned participants into one of four dietary supplementation interventions: no nutritional supplement control (CON), carbohydrate placebo (PLA), moderate whey protein [20 g additional per day (MOD)] or high whey protein [60 g additional per day (HIGH)]. All data was collected in week 12 of training immediately before, after (<1-h), 24-h (24 h-POST) after and 40-h (40 h-POST) after a standardized British Army BT load carriage test. The study was approved by the U.K. Ministry of Defence Research Ethics Committee (1076/MODREC/20) and was conducted in accordance with the principles defined in the Declaration of Helsinki 1 as adopted at the 64th WMA General Assembly at Fortaleza, Brazil in October 2013. Ninety-nine men and 23 women [CON: men = 19, women = 7; PLA: men = 27, women = 5; MOD: men = 27, women = 4; HIGH: men = 26, women = 7 (mean ± standard deviation: age: 21.3 ± 3.5 years., height: 174.8 ± 8.4 cm, body mass 75.4 ± 12.2 kg)] were recruited in week one of training at the Army Training Centre, Pirbright (Surrey, United Kingdom). All participants passed their medical assessment prior to starting BT. Sample size was based on *a priori* power analysis using G*power (Dusseldorf, V 3.1). It was determined that 52 participants were required to replicate a partial eta squared (*η_p_*^2^) of 0.06 (medium) using an *α* = 0.05 and *β* = 0.95 for a within-subjects and-between-group analysis to determine differences in the primary muscle damage marker, myoglobin. A second power calculation was completed for the estimated sample size required to detect a within-subjects and-between-group difference in muscle function. It was determined that 16 participants were required to replicate a *η_p_*^2^ of 0.23 (large) using an *α* = 0.05 and *β* = 0.95. This study was registered with ClinicalTrials.gov, U.S. National Institutes (identifier: NCT05998603).

### 2.2 Supplementation, dietary intake, and nitrogen balance

The study design is described in greater detail including the supplementation, dietary intake, and nitrogen balance methodologies, in the accompanying article of this dual submission (31). Participants were administered the supplements each weekday evening from the start of week 3 until week 12 between 20:00 and 21:00 h in powder form mixed with water by members of the research team. The supplements were isocaloric to isolate the effects of the additional protein intake. The nutritional breakdown of each supplement, the dietary intake and nitrogen balance of participants is shown in the Supplementary materials.

### 2.3 Load carriage test

Participants underwent a standardized British Army BT load carriage test as part of their formative Role Fitness Test in week 12 of BT and supervised by Army physical training instructors. The physical activity of each group before and after the load carriage test was also standardized with all participants instructed by their platoon staff during the 14-week British Army course. As well as standardized activities, participants also shared accommodation, limiting the variation in physical activity between groups. The test was completed with participants wearing standard Army uniform and boots with helmets carried in their Bergen. The test comprised of two parts: (i) 4 km loaded march carrying 20 kg in total (webbing: 5.5 kg, Bergen: 9 kg, rifle: 4.5 kg) at 4.8 kph, (ii) a 2 km run carrying 15 kg in total (webbing: 5.5 kg, patrol sack: 4 kg, rifle: 4.5 kg). Participants were required to complete the test faster than the standard required for their role (<15–17 min dependent on role) to pass BT.

### 2.4 Muscle function

Vertical jump performance was used to assess muscle function at each timepoint using a vertical jump meter (Takei, Scientific instruments, Niigata city, Japan) (5, 7). Participants were instructed to jump as high as possible three times with their hands placed on their hips to prevent upper limb assistance (32). Participant’s completed a fourth jump if their third attempt was their highest to reduce the chance of a learning affect. The participant’s best score was recorded. The test–retest reliability of *r* ≥ 0.90 has been reported for this performance test (33). Footwear and clothing were standardized across all participants for each vertical jump test.

### 2.5 Muscle damage

The concentrations of urinary myoglobin, titin and 3-methylhistidine (3-MH) were measured from a spot 60 mL urine sample at each timepoint (pre, immediately-post, 24-h-post and 40-h-post). Urine samples were refrigerated at 4°C immediately on receipt prior to being pipetted into three 5 mL cryotubes and stored at −80°C. The samples were then transferred to Anglia Ruskin University, Cambridge, UK for analysis. The concentrations of myoglobin (Abcam, Cambridge, United Kingdom), titin (MyBioSource, San Diego, United States) and 3-MH (Abbexa, Cambridge, United Kingdom), were quantified by an Enzyme-Linked Immunosorbent Assay (ELISA) following the manufacturer’s protocol (34–36). The inter and intra assay coefficient of variation (CV) was 6.4 and 3.4% for myoglobin, 9.9 and 9.7% for 3-MH and 8.1 and 7.8% for titin, respectively.

### 2.6 Muscle soreness

While standing, participants were asked to record their perceived muscle soreness using a 0–10 Likert scale (5). Participants were also asked to record their subjective muscle soreness before, immediately after, 24 h-POST after and 40 h-POST after the load carriage test.

### 2.7 Statistical analysis

Statistical analyses were carried out using the Statistical Package for Social Sciences (v26, IBM, Armock, New York, United States) with significance set at *p* ≤ 0.05. Data were assessed for normality visually and using the Shapiro–wilk test. Mixed model Analyses of Covariances (ANCOVAs) were used to examine changes in muscle function, muscle damage and subjective measures at each timepoint and between supplementation groups. In all instances, the pre-measures were used as covariates. All post-hoc analyses were undertaken using an adjusted Bonferroni *post hoc* test. Data were presented as mean ± standard deviation with partial eta squared (*ƞ_p_*^2^) effect sizes to denote small (0.01), medium (0.06), and large (≥0.14) effects (32).

## 3 Results

### 3.1 Muscle function and soreness

The descriptive data for muscle function and muscle soreness are shown in Table 1. After adjusting for PRE jump height, there was no group interaction at POST [*F*(3, 83) = 0.402, *p* = 0.752, *ƞ_p_*^2^ = 0.014] or 40-POST [*F*(1, 83) = 0.040, *p* = 0.989, *ƞ_p_*^2^ = 0.001] but there was at 24 h-POST [*F*(1, 83) = 2.847, *p* = 0.042, *ƞ_p_*^2^ = 0.093]. *Post hoc* analysis revealed greater jump height in PLA compared to HIGH at 24 h-POST (*p* = 0.037). After adjusting for PRE muscle soreness, there was no group interaction at POST [*F*(3, 86) = 0.618, *p* = 0.605, *ƞ_p_*^2^ = 0.021], 24 h-POST [*F*(3, 86) = 1.658, *p* = 0.182, *ƞ_p_*^2^ = 0.055] or 40 h-POST [*F*(3, 86) = 1.151, *p* = 0.333, *ƞ_p_*^2^ = 0.039].

**Table 1:** Muscle soreness and jump height pre-and-post the loaded march test in each group.

Measure	Group	PRE	POST	24H-POST	40H-POST
Muscle soreness	CON	2 ± 1	4.0 ± 1	3 ± 2	2 ± 2
	PLA	2 ± 1	3.0 ± 1	2 ± 1	3 ± 2
	MOD	2 ± 2	3.0 ± 1	2 ± 2	2 ± 1
	HIGH	2 ± 2	3 ± 2	2 ± 2	2 ± 2
Jump height (cm)	CON	34.7 ± 10.8	38.6 ± 6.8	39.0 ± 7.7	36.0 ± 7.5
	PLA	36.3 ± 6.7	37.0 ± 6.3	39.8 ± 6.0^d^	37.0 ± 6.2
	MOD	38.3 ± 5.2	37.1 ± 5.9	40.0 ± 6.5	36.0 ± 5.6
	HIGH	36.2 ± 6.0	37.7 ± 8.4	35.7 ± 7.4^b^	35.7 ± 6.4

Data presented as mean ± SD. CON, control; PLA, placebo; MOD, moderate protein; HIGH, high protein; PRE, pre-march; POST, <1 h post march; 24 h-POST, 24 h post; 40 h-POST, 40 h post; cm, centimeters. Different vs. CON, ^b^different vs. PLA, different vs. MOD and ^d^different vs. HIGH.

### 3.2 Muscle damage markers

**Figure 1:**
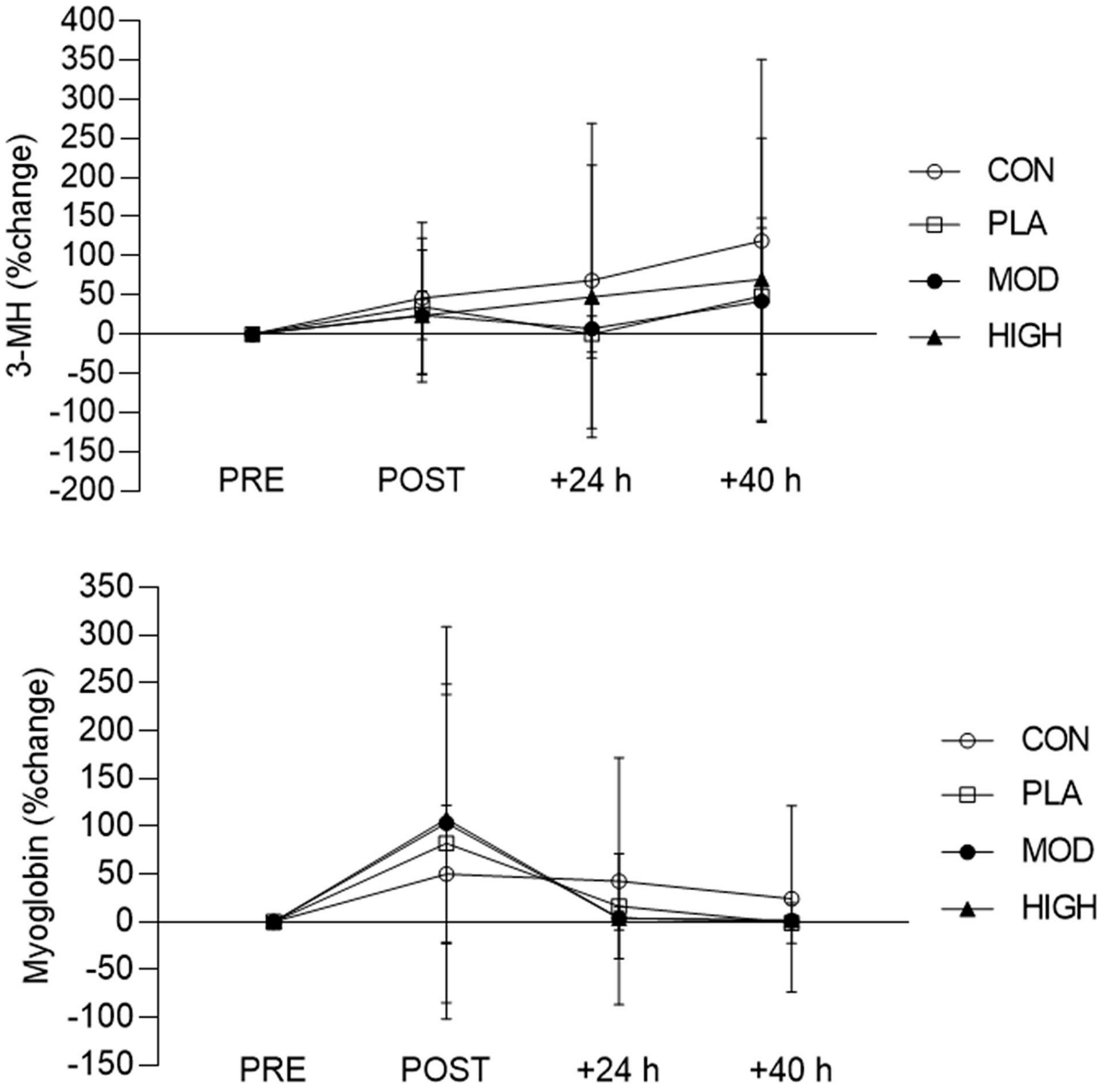
Percentage (%) change in urinary 3-MH and myoglobin for each group pre and post the loaded march test. Data presented as mean ± SD. CON, control; PLA, placebo; MOD, moderate protein; HIGH, high protein; PRE, pre-march; POST, <1 h post march; 24-POST, 24 h post; 40H-POST, 40 h post.

The descriptive data for each marker of muscle damage is shown in Table 2. The % change data for 3-MH and myoglobin are also shown in Figure 1. After adjusting for PRE-3-MH concentrations, there was no group interaction at POST [*F*(3, 52) = 0.970, *p* = 0.414, *ƞ_p_*^2^ = 0.053], 24 h-POST [*F*(3, 52) = 0.298, *p* = 0.826, *ƞ_p_*^2^ = 0.017] or 40 h-POST [*F*(3, 52) = 0.353, *p* = 0.787, *ƞ_p_*^2^ = 0.020]. After controlling for PRE myoglobin concentrations, there was no group interaction at POST [*F*(3, 54) = 0.713, *p* = 0.548, *ƞ_p_*^2^ = 0.038] or 24 h-POST [*F*(3, 54) = 1.025, *p* = 0.389, *ƞ_p_*^2^ = 0.054] or 40 h-POST [*F*(3, 54) = 1.069, *p* = 0.370, *ƞ_p_*^2^ = 0.056].

**Table 2:** The concentration for each marker of muscle damage is shown for each group at each timepoint.

Measure	Group	PRE	POST	24H-POST	40H-POST
3-MH (nmol⸱mL^−1^)	CON	185.3 ± 109.1	207.3 ± 100.9	195.6 ± 88.8	263.4 ± 91.6
	PLA	240.5 ± 85.1	277.6 ± 70.3	228.3 ± 67.6	294.1 ± 47.4
	MOD	217.3 ± 66.9	266.6 ± 81.3	223.1 ± 72.1	264.8 ± 57.5
	HIGH	221.8 ± 110.9	242.5 ± 117.7	234.9 ± 81.5	265.3 ± 76.8
Myoglobin (ng⸱mL^−1^)	CON	87.0 ± 20.1	128.9 ± 60.5	129.7 ± 129.5	105.3 ± 77.1
	PLA	87.6 ± 20.7	151.7 ± 129.0	97.9 ± 41.4	83.9 ± 12.2
	MOD	82.4 ± 4.5	163.5 ± 162.2	85.7 ± 11.2	83.1 ± 4.0
	HIGH	80.8 ± 1.5	167.5 ± 104.8	83.9 ± 5.6	82.3 ± 3.9

Data presented as mean ± SD. CON, control; PLA, placebo; MOD, moderate protein; HIGH, high protein; PRE, pre-march; POST, <1 h post march; 24-POST, 24 h post; 40H-POST, 40 h post; ng, nanograms; nm, nanomole; ml, milliliter.

## 4 Discussion

The aim of this study was to establish the impact of the MOD (20 g) and HIGH (60 g) protein bolus prior to sleep on acute muscle recovery following a load carriage test in British Army recruits. The key finding was that the changes in muscle function, muscle soreness and muscle damage were similar between groups. Therefore, the data does not support the use of protein supplementation to support acute muscle recovery during British Army BT irrespective of relative quantity. Research in healthy young adults has shown protein supplementation can be effective to accelerate muscle function recovery (20, 27, 37) and attenuate muscle soreness (21, 27) and exercise-induced muscle damage (16–19) compared to a control or placebo. However, research pertinent to military training is limited (38).

Protein feeding post-exercise can attenuate EIMD by increasing the bioavailability of essential amino acids necessary for MPS and skeletal muscle remodeling, which in turn, can support functional muscle recovery (17). Consuming 20–40 g of protein post-exercise maximizes the MPS response (39, 40). Based on habitual protein intakes (2), an additional 20 or 60 g bolus dose increased total daily protein intake to the upper limit and beyond the recommendation for protein intake during military training (41). The release of essential amino acids into the circulation, in particular L-leucine, is stimulatory for MPS in the post-exercise recovery period (42). L-leucine has been shown to independently upregulate MPS by activating the mammalian target of rapamycin complex-1 (42). Generally, it is recognized that 8–10 g (1.8–2.0 g leucine) of essential amino acids maximizes MPS with whey protein specifically leading to a rapid rise in blood amino acid concentrations and therefore MPS (43, 44). Nonetheless, it is generally recognized that the total daily intake of protein and whole-body protein balance (i.e., nitrogen balance) are key determinants compared to the timing of protein intake in the context of muscle recovery (45). In the context of this study, the data suggests consuming additional calories, either in the form of CHO or protein attenuated EIMD (Figure 1). This is, in part, is in agreement with previous work which has shown CHO or protein supplementation to accelerate muscle recovery following load carriage compared to a control condition (7). Mechanistically, this may explain the protective effects of protein or CHO supplementation on injury risk during military training (9). It is recognized that CHO feeding attenuates muscle protein breakdown via insulin secretion (19), which may explain the attenuated EIMD observed in this study (Figure 1). Nevertheless, it is acknowledged that in our study, no statistical differences were detected between groups for muscle function. Nonetheless, the attenuation of EIMD with supplementation suggests consuming additional energy, in the form of CHO or protein, following arduous military training activities may be beneficial for muscle recovery and therefore could impact subsequent musculoskeletal injury risk.

The dietary intakes of participants are shown in the accompanying article of this dual submission (31) and in the Supplementary materials. As hypothesized, protein supplementation increased total daily protein intakes for the MOD (1.71 ± 0.29 g⸱kg^−1^⸱day^−1^) and HIGH protein groups (2.16 ± 0.50 g⸱kg^−1^⸱day^−1^) compared to PLA (1.31 ± 0.29 g⸱kg^−1^⸱day^−1^) and CON conditions (1.17 ± 0.24 g⸱kg^−1^⸱day^−1^) (Supplementary Table S2). This resulted in a significantly greater positive nitrogen balance in the HIGH (10.7 ± 3.5 g⸱day^−1^) and MOD (2.9 ± 4.6 g⸱day^−1^) protein conditions compared to the PLA (2.3 ± 3.8 g⸱day^−1^) and CON groups (2.6 ± 2.7 g⸱day^−1^) (Supplementary Table S3). The changes in urinary muscle damage markers, indicated that the load carriage test elicited some degree of EIMD (Figure 1). The observed elevation in 3-MH and myoglobin were similar to those reported following muscle-damaging exercise in athletes (34, 46, 47). 3-MH concentrations gradually increased in all groups before peaking at 40 h post-exercise, while myoglobin increased rapidly, peaking in concentration within 1 h-post load carriage. Despite no statistically significant difference between groups at any timepoint, 3-MH and myoglobin concentrations remained elevated in the control group compared to the supplementation groups. The increased EIMD as indicated by urinary 3-MH and myoglobin in this study did not result in improved muscle function and/or reduced muscle soreness following the load carriage test (Table 1).

The similarity in the changes in muscle function between groups (Table 1) maybe due to several reasons. Vertical jump height is reflective of lower-body performance, specific to military training and has been used by others in this population (5, 7, 32, 33). Vertical jump height testing is also considered a surrogate measure of maximal voluntary contraction to estimate EIMD (22). Previous studies assessing muscle function following a load carriage test in military recruits have demonstrated lower jump height (−5 to −8%) within 1 h (5, 7). However, in this study, there was no detrimental impact of load carriage on jump height. It should be noted that participants in previous research (5) completed the load carriage test in week 12, similar to this cohort. However, a key difference between the present study and others (5, 7, 24) is the distance participants undertook and load carried for the load carriage test. The distance was substantially lower in the present study (4 km load carriage) compared to O’Leary et al. (5) (10 km) and Fallowfield et al. (7) (19 km). The maximum mass carried by participants was less in the present study (15 kg) compared to studies by Fallowfield et al. (7) (31 kg) and Blacker et al. (6) (25 kg), who observed a − 15% and − 8% reduction in MVC and vertical jump height, respectively. Indeed, it is possible that improvements in recruit physical fitness during BT were sufficient to mitigate against muscle function loss following the current load carriage in week 12. For example, individuals who engage in novel exercise regimes experience greater degrees of EIMD, including muscle function loss, compared to trained individuals (22). As the load carriage test was undertaken in week 12, it may be that the participants were already sufficiently trained in load carriage to attenuate any notable loss in muscle function. Furthermore, a combination of the shorter load carriage test distance and the lower weight carried may explain the limited impact of protein supplementation observed. The lack of muscle function loss as measured by vertical jump height would support this premise.

## 5 Strength and limitations

There are several strengths of this research that should be highlighted. Firstly, this research is novel with currently limited data in nutritional interventions during British Army BT. This study has high ecological validity, included men and women, and was sufficiently powered. An additional strength is the randomization of participants and the isolated effects of protein using isocaloric conditions. Finally, adherence to supplementation should also be acknowledged as a strength due to the research team supervising each supplementation condition. Firstly, total daily protein intake was estimated using a self-report food diary which is known to underestimate dietary intake (48). Therefore, participants may have habitually consumed higher protein intakes which would be expected to limit the impact of protein supplementation between groups (49). However, all participants followed a standardized military diet which was well controlled. Secondly, the lack of change in muscle function following the load carriage test may have been due to the demands of the load carriage test not being high enough, whereby the test distance was not long enough or the weight carried by participants was not substantial enough compared to previous studies (5–7, 24). Nevertheless, this test is reflective of British Army training and therefore the results can be considered to be ecologically valid due to their real-world implications. However, if previous test conditions were employed or were to be reinstated to BT then potential differences may be observed. Although an increase in EIMD was observed as measured by urinary myoglobin and 3-MH, there was substantial inter-individual variability in each muscle damage marker response which limits the comparison between the supplementation groups. However, the intra and inter assay variability for each marker was within an acceptable range (<10%) (Myoglobin: inter = 6.4% and intra = 3.4%; 3-MH: inter = 9.7% and intra = 9.9%; titin: inter = 8.1% and intra = 7.8%). Although participants followed a standardized military training program, it was not possible to instruct participants to rest before and between study measurements, potentially influencing the measures of recovery. It should be acknowledged that a strength of the study was that women were included but most participants were men, therefore extrapolating the findings to women should be done with caution, particularly as women experience greater muscular fatigue following load carriage (5). Finally, this study is the result of survival bias as only participants who completed training were able to complete the study.

## 6 Conclusion

Pre-sleep protein supplementation increased total daily protein intake and nitrogen balance in British Army recruits. However, protein supplementation (irrespective of dose) did not improve muscle recovery following a load carriage test in week 12 of British Army BT with similar changes in muscle function, perceived soreness, and damage between groups. The lack of muscle function loss following the load carriage test likely limited the influence of protein supplementation on markers of muscle recovery. Protein supplementation above protein intakes of 1.2 g⸱kg^−1^⸱day^−1^ following load carriage over similar distances (4 km) and carrying similar loads (15–20 kg) does not appear to be warranted. The data infers that both protein and CHO supplementation attenuated EIMD with high concentrations of myoglobin and 3-MH observed post-exercise, but this did not improve muscle function and/or muscle soreness in the days following the load carriage test. Nonetheless this result does suggest consuming additional calories in the form of CHO or protein following arduous military activities may be beneficial for acute muscle recovery by attenuating EIMD. This may be more physiologically relevant following load carriage over longer distances and/or carrying heavier loads. As this was the first study of this nature in this population, further research is warranted to corroborate these findings and explore whether specific nutritional supplementation timing (i.e., immediately post-exercise) may confer individual recovery benefits and if this ultimately reduces injury risk.

## Data availability statement

The raw data supporting the conclusions of this article will be made available by the authors, without undue reservation.

## Ethics statement

The studies involving humans were approved by the Ministry of Defence Research Ethics Committee. The studies were conducted in accordance with the local legislation and institutional requirements. The participants provided their written informed consent to participate in this study.

## Author contributions

SC: Conceptualization, Data curation, Formal analysis, Investigation, Methodology, Writing – original draft, Writing – review & editing. JR: Conceptualization, Funding acquisition, Methodology, Writing – review & editing, Formal analysis. ARo: Conceptualization, Investigation, Writing – review & editing. HO: Investigation, Methodology, Writing – review & editing. RI: Conceptualization, Funding acquisition, Investigation, Supervision, Writing – review & editing. LSm: Conceptualization, Funding acquisition, Investigation, Supervision, Writing – review & editing. HC: Formal analysis, Methodology, Writing – review & editing. LSt: Investigation, Writing – review & editing. ARa: Conceptualization, Funding acquisition, Methodology, Writing – review & editing, Investigation.

## References

[1] O’Leary TJ Saunders SC Mcguire SJ Venables MC Izard RM. Sex differences in training loads during British Army basic training. Med Sci Sports Exerc. (2018) 50:2565–74. doi: 10.1249/MSS.0000000000001716 30048410

[2] Chapman S Rawcliffe AJ Izard R Jacka K Tyson H Smith L. Dietary intake and nitrogen balance in British Army infantry recruits undergoing basic training. Nutrients. (2020) 12:2125. doi: 10.3390/nu12072125 32709021 PMC7400853

[3] Edwards V Myers S Wardle S Siddall A Powell S Needham-Beck S. Nutrition and physical activity in British Army officer cadet training part 2—daily distribution of energy and macronutrient intake. Int J Sport Nutr Exerc Metab. (2022) 32:204–13. doi: 10.1123/ijsnem.2021-0191 35294923

[4] Chapman S Roberts J Smith L Rawcliffe A Izard R. Sex differences in dietary intake in British Army recruits undergoing phase one training. J Int Soc Sports Nutr. (2019) 16:59. doi: 10.1186/s12970-019-0327-2, PMID: 31823790 PMC6905050

[5] O’Leary TJ Saunders SC McGuire SJ Izard RM. Sex differences in neuromuscular fatigability in response to load carriage in the field in British Army recruits. J Sci Med Sport. (2018) 21:591–5. doi: 10.1016/j.jsams.2017.10.018, PMID: 29100827

[6] Blacker SD Williams NC Fallowfield JL Bilzon JLJ Willems MET. Carbohydrate vs protein supplementation for recovery of neuromuscular function following prolonged load carriage. J Int Soc Sports Nutr. (2010) 7:1–11. doi: 10.1186/1550-2783-7-2 20157419 PMC2821364

[7] Fallowfield JL Blacker SD Willems MET Davey T Layden J. Neuromuscular and cardiovascular responses of Royal Marine recruits to load carriage in the field. Appl Ergon. (2012) 43:1131–7. doi: 10.1016/j.apergo.2012.04.003, PMID: 22575491

[8] Blacker SD Fallowfield JL Bilzon JLJ Willems MET. Neuromuscular function following prolonged load carriage on level and downhill gradients. Aviat Space Environ Med. (2010) 81:745–53. doi: 10.3357/ASEM.2659.2010, PMID: 20681234

[9] McGinnis K McAdam J Lockwood C Young K Roberts M Sefton J. Impact of protein and carbohydrate supplementation on musculoskeletal injuries in Army initial entry training soldiers. Nutrients. (2018) 10:1938. doi: 10.3390/nu10121938, PMID: 30563273 PMC6315558

[10] O’Leary TJ Wardle SL Rawcliffe AJ Chapman S Mole J Greeves JP. Understanding the musculoskeletal injury risk of women in combat: the effect of infantry training and sex on musculoskeletal injury incidence during British Army basic training. BMJ Mil Health. (2020) 169:57–61. doi: 10.1136/jramc-2019-001347 32111683

[11] Sharma J Greeves JP Byers M Bennett AN Spears IR. Musculoskeletal injuries in British Army recruits: a prospective study of diagnosis-specific incidence and rehabilitation times. BMC Musculoskelet Disord. (2015) 16:106. doi: 10.1186/s12891-015-0558-6, PMID: 25935751 PMC4443544

[12] Sharma J Heagerty R Dalal S Banerjee B Booker T. Risk factors associated with musculoskeletal injury: a prospective study of British infantry recruits. CRR. (2018) 15:50–8. doi: 10.2174/1573397114666180430103855 29708075

[13] McAdam J McGinnis K Ory R Young K Frugé AD Roberts M. Estimation of energy balance and training volume during Army initial entry training. J Int Soc Sports Nutr. (2018) 15:55. doi: 10.1186/s12970-018-0262-7, PMID: 30486851 PMC6264031

[14] McAdam JS Lyons KD Beck DT Haun CT Romero MA Mumford PW. Whey protein supplementation effects on body composition, performance, and blood biomarkers during Army initial entry training. Front Nutr. (2022) 9:807928. doi: 10.3389/fnut.2022.807928 35330708 PMC8940516

[15] Tipton KD Hamilton DL Gallagher IJ. Assessing the role of muscle protein breakdown in response to nutrition and exercise in humans. Sports Med. (2018) 48:53–64. doi: 10.1007/s40279-017-0845-5, PMID: 29368185 PMC5790854

[16] Bongiovanni T Genovesi F Nemmer M Carling C Alberti G Howatson G. Nutritional interventions for reducing the signs and symptoms of exercise-induced muscle damage and accelerate recovery in athletes: current knowledge, practical application and future perspectives. Eur J Appl Physiol. (2020) 120:1965–96. doi: 10.1007/s00421-020-04432-3, PMID: 32661771

[17] Howatson G Hoad M Goodall S Tallent J Bell PG French DN. Exercise-induced muscle damage is reduced in resistance-trained males by branched chain amino acids: a randomized, double-blind, placebo controlled study. J Int Soc Sports Nutr. (2012) 9:20. doi: 10.1186/1550-2783-9-20, PMID: 22569039 PMC3395580

[18] Harty PS Cottet ML Malloy JK Kerksick CM. Nutritional and supplementation strategies to prevent and attenuate exercise-induced muscle damage: a brief review. Sports Med. (2019) 5:1. doi: 10.1186/s40798-018-0176-6, PMID: 30617517 PMC6323061

[19] Owens DJ Twist C Cobley JN Howatson G Close GL. Exercise-induced muscle damage: what is it, what causes it and what are the nutritional solutions? Eur J Sport Sci. (2019) 19:71–85. doi: 10.1080/17461391.2018.1505957 30110239

[20] Davies R Carson B Jakeman P. The effect of whey protein supplementation on the temporal recovery of muscle function following resistance training: a systematic review and Meta-analysis. Nutrients. (2018) 10:221. doi: 10.3390/nu10020221, PMID: 29462923 PMC5852797

[21] Jackman SR Witard OC Jeukendrup AE Tipton KD. Branched-chain amino acid ingestion can ameliorate soreness from eccentric exercise. Med Sci Sports Exerc. (2010) 42:962–70. doi: 10.1249/MSS.0b013e3181c1b798 19997002

[22] Chalchat E Gaston AF Charlot K Peñailillo L Valdés O Tardo-Dino PE. Appropriateness of indirect markers of muscle damage following lower limbs eccentric-biased exercises: a systematic review with meta-analysis. PLoS One. (2022) 17:e0271233. doi: 10.1371/journal.pone.0271233 35834532 PMC9282447

[23] Pasiakos SM McClung HL Margolis LM Murphy NE Lin GG Hydren JR. Human muscle protein synthetic responses during weight-bearing and non-weight-bearing exercise: a comparative study of exercise modes and recovery nutrition. PLoS One. (2015) 10:e0140863. doi: 10.1371/journal.pone.0140863 26474292 PMC4608805

[24] Flakoll PJ Judy T Flinn K Carr C Flinn S. Postexercise protein supplementation improves health and muscle soreness during basic military training in marine recruits. J Appl Physiol. (2004) 96:951–6. doi: 10.1152/japplphysiol.00811.2003, PMID: 14657039

[25] Jimenez-Flores R Heick J Davis SC Hall KG Schaffner A. A comparison of the effects of a high carbohydrate vs. a higher protein milk supplement following simulated mountain skirmishes. Mil Med. (2012) 177:723–31. doi: 10.7205/MILMED-D-11-00396 22730850

[26] Trommelen J VanLoon LJC. Pre-sleep protein ingestion to improve the skeletal muscle adaptive response to exercise training. Nutrients. (2016) 8:763. doi: 10.3390/nu8120763, PMID: 27916799 PMC5188418

[27] Abbott W Brett A Cockburn E Clifford T. Presleep casein protein ingestion: acceleration of functional recovery in professional soccer players. Int J Sports Physiol Perform. (2019) 14:385–91. doi: 10.1123/ijspp.2018-0385 30204517

[28] Ormsbee MJ Saracino PG Morrissey MC Donaldson J Rentería LI McKune AJ. Pre-sleep protein supplementation after an acute bout of evening resistance exercise does not improve next day performance or recovery in resistance trained men. J Int Soc Sports Nutr. (2022) 19:164–78. doi: 10.1080/15502783.2022.2036451, PMID: 35599912 PMC9116400

[29] Larsen MS Clausen D Jørgensen AA Mikkelsen UR Hansen M. Presleep protein supplementation does not improve recovery during consecutive days of intense endurance training: a randomized controlled trial. Int J Sport Nutr Exerc Metab. (2019) 29:426–34. doi: 10.1123/ijsnem.2018-0286, PMID: 30632413

[30] Apweiler E Wallace D Stansfield S Allerton DM Brown MA Stevenson EJ. Pre-bed casein protein supplementation does not enhance acute functional recovery in physically active males and females when exercise is performed in the morning. Sports. (2019) 7:5. doi: 10.3390/sports7010005 PMC6359469 30597848

[31] Chapman S Roberts J Roberts A Ogden H Izard R Smith L. Pre-sleep protein supplementation does not improve performance, body composition and recovery in British Army recruits (part 1). In submission (2023). doi: 10.3389/fnut.2023.1262044 PMC10748761 38144428

[32] Fortes MB Diment BC Greeves JP Casey A Izard R Walsh NP. Effects of a daily mixed nutritional supplement on physical performance, body composition, and circulating anabolic hormones during 8 weeks of arduous military training. Appl Physiol Nutr Metab. (2011) 36:967–75. doi: 10.1139/h11-124 22111592

[33] Carswell AT Oliver SJ Wentz LM Kashi DS Roberts R Tang JCY. Influence of vitamin D supplementation by sunlight or Oral D3 on exercise performance. Med Sci Sports Exerc. (2018) 50:2555–64. doi: 10.1249/MSS.0000000000001721, PMID: 30048414 PMC6282681

[34] Lindsay A Lewis J Scarrott C Draper N Gieseg SP. Changes in acute biochemical markers of inflammatory and structural stress in rugby union. J Sports Sci. (2015) 33:882–91. doi: 10.1080/02640414.2014.971047 25358055

[35] Yasuda J Gomi T Kotemori A Yokoyama Y. Breakfast before resistance exercise lessens urinary markers of muscle protein breakdown in young men: a crossover trial. Nutrition. (2021) 83:111088. doi: 10.1016/j.nut.2020.111088 33418493

[36] Kanda K Sakuma J Akimoto T Kawakami Y Suzuki K. Detection of titin fragments in urine in response to exercise-induced muscle damage. Vlahou A, editor. PLoS One (2017) 12:e0181623. doi: 10.1371/journal.pone.0181623 28727760 PMC5519174

[37] West D Abou Sawan S Mazzulla M Williamson E Moore D. Whey protein supplementation enhances whole body protein metabolism and performance recovery after resistance exercise: a double-blind crossover study. Nutrients. (2017) 9:735. doi: 10.3390/nu9070735, PMID: 28696380 PMC5537849

[38] Chapman S Chung HC Rawcliffe AJ Izard R Smith L Roberts JD. Does protein supplementation support adaptations to arduous concurrent exercise training? A systematic review and Meta-analysis with military based applications. Nutrients. (2021) 13:1416. doi: 10.3390/nu13051416, PMID: 33922458 PMC8145048

[39] Macnaughton LS Wardle SL Witard OC McGlory C Hamilton DL Jeromson S. The response of muscle protein synthesis following whole-body resistance exercise is greater following 40 g than 20 g of ingested whey protein. Physiol Rep. (2016) 4:e12893. doi: 10.14814/phy2.12893, PMID: 27511985 PMC4985555

[40] Witard O Wardle S Macnaughton L Hodgson A Tipton K. Protein considerations for Optimising skeletal muscle mass in healthy Young and older adults. Nutrients. (2016) 8:181. doi: 10.3390/nu8040181, PMID: 27023595 PMC4848650

[41] Pasiakos SM Austin KG Lieberman HR Askew EW. Efficacy and safety of protein supplements for U.S. armed forces personnel: consensus statement. J Nutr. (2013) 143:1811S–4S. doi: 10.3945/jn.113.176859, PMID: 24027189

[42] Zaromskyte G Prokopidis K Ioannidis T Tipton KD Witard OC. Evaluating the leucine trigger hypothesis to explain the post-prandial regulation of muscle protein synthesis in Young and older adults: a systematic review. Front Nutr. (2021) 8:685165. doi: 10.3389/fnut.2021.685165 34307436 PMC8295465

[43] Burd NA Tang JE Moore DR Phillips SM. Exercise training and protein metabolism: influences of contraction, protein intake, and sex-based differences. J Appl Physiol. (2009) 106:1692–701. doi: 10.1152/japplphysiol.91351.2008, PMID: 19036897

[44] Reidy PT Rasmussen BB. Role of ingested amino acids and protein in the promotion of resistance exercise–induced muscle protein anabolism. J Nutr. (2016) 146:155–83. doi: 10.3945/jn.114.203208, PMID: 26764320 PMC4725426

[45] Pasiakos SM Lieberman HR McLellan TM. Effects of protein supplements on muscle damage, soreness and recovery of muscle function and physical performance: a systematic review. Sports Med. (2014) 44:655–70. doi: 10.1007/s40279-013-0137-7, PMID: 24435468

[46] Colombani PC Kovacs E Frey-Rindova P Frey W Langhans W Arnold M. Metabolic effects of a protein-supplemented carbohydrate drink in Marathon runners. Int J Sport Nutr Exerc Metab. (1999) 9:181–201. doi: 10.1123/ijsn.9.2.181, PMID: 10362454

[47] Coso JD González-Millán C Salinero JJ Abián-Vicén J Soriano L Garde S. Muscle damage and its relationship with muscle fatigue during a half-Iron triathlon. PLoS One. (2012) 7:e43280. doi: 10.1371/journal.pone.0043280, PMID: 22900101 PMC3416828

[48] Magkos F Yannakoulia M. Methodology of dietary assessment in athletes: concepts and pitfalls. Curr Opin Clin Nutr Metab Care. (2003) 6:539–49. doi: 10.1097/00075197-200309000-00007 12913671

[49] Morton RW Murphy KT McKellar SR Schoenfeld BJ Henselmans M Helms E. A systematic review, meta-analysis and meta-regression of the effect of protein supplementation on resistance training-induced gains in muscle mass and strength in healthy adults. Br J Sports Med. (2017) 52:376–84. doi: 10.1136/bjsports-2017-097608 28698222 PMC5867436

