# Supplementary material for: Pre-sleep protein supplementation does not improve recovery from load carriage in British Army recruits (part 2)
Source: Front Nutr. 2023 Nov 30;10:1264042. doi: 10.3389/fnut.2023.1264042 (PMC10733965; doi:10.3389/fnut.2023.1264042)
Supplement: Supplementary file 1 [file Data_Sheet_1.docx]

Supplementary Material

The influence of protein supplementation prior to sleep on load carriage recovery in British Army recruits

**Shaun Chapman*, Justin Roberts, Andrew Roberts, Henry Ogden, Rachel Izard, Lee Smith, Lauren Stuszczak, Alex Rawcliffe**

*** Correspondence:** Shaun Chapman: shaun.chapman101@mod.gov.uk

# Supplementary Tables

Supplementary Table 1. The energy and macronutrient content of each supplement. HIGH=high protein, MOD=moderate protein, PLA=-placebo.

| **Supplement** | **Energy (kcal)** | **CHO (g)** | **Protein (g)** | **Fat (g)** |
| --- | --- | --- | --- | --- |
| HIGH | 296.0 | 8.0 | 60.0 | 2.5 |
| MOD | 286.0 | 48.0 | 20.0 | 1.5 |
| PLA | 286.0 | 67.0 | 2.2 | 1.1 |

Supplementary Table 2. Daily dietary intake in each group. Data shown as mean ± standard deviation. g=grams, kg=kilograms, d=day, CHO=carbohydrate, a=different vs. CON, b=different vs. PLA, c=different vs. MOD and d=different vs. HIGH.

| **Group** | **Energy (kcal)** | **CHO (g)** | **Protein (g)** | **Fat (g)** |
| --- | --- | --- | --- | --- |
| CON | 2146.72 ± 393.46^b, c, d^ | 246.49 ± 49.48^b^ | 91.95 ± 18.22^c,d^ | 88.06 ± 22.24 |
| PLA | 2530.80 ± 617.04^a^ | 331.63 ± 84.38^a, d^ | 98.24 ± 21.20^c, d^ | 90.18 ± 28.04 |
| MOD | 2632.45 ± 591.63^a^ | 322.30 ± 82.31 | 125.09 ± 26.32^a, b, d^ | 94.23 ± 27.72 |
| HIGH | 2648.17 ± 475.09^a^ | 278.80 ± 74.78^b^ | 156.95 ± 17.44^a, b, c^ | 100.66 ± 22.73 |
| **Group** | **Energy (kcal⸱kg^-1^⸱day^-1^)** | **CHO (g⸱kg^-1^⸱day^-1^)** | **Protein (g⸱kg^-1^⸱day^-1^)** | **Fat (g⸱kg^-1^⸱day^-1^)** |
| CON  PLA  MOD  HIGH | 27.59 ± 6.28  34.00 ± 9.11  36.15 ± 11.31  36.17 ± 8.79 | 3.18 ± 0.86b  4.48 ± 1.31a,c  4.44 ± 1.57b,d  3.80 ± 1.15c | 1.17 ± 0.24c,d  1.31 ± 0.29c,d  1.71 ± 0.48a,b,d  2.16 ± 0.50a,b,c | 1.13 ± 0.31  1.20 ± 0.38  1.29 ± 0.48  1.38 ± 0.37 |

Supplementary Table 3. Nitrogen balance in each group. N=nitrogen, g=grams, kg=kilograms, CON=control, PLA=placebo, MOD=moderate, HIGH=high, ^a^=difference vs. CON, ^b^=difference vs. PLA, ^c^=difference vs. MOD and ^d^=difference vs. HIGH.

| **Group** | **N excretion (g)** | **N intake (g)** | **N balance (g)** |
| --- | --- | --- | --- |
| CON | 11.5 ± 2.1 | 14.1 ± 1.8^d^ | 2.6 ± 2.7^d^ |
| PLA | 11.9 ± 2.8 | 14.2 ± 1.8^d^ | 2.3 ± 3.8^d^ |
| MOD | 13.7 ± 3.2 | 16.6 ± 2.9^d^ | 2.9 ± 4.6^d^ |
| HIGH | 14.4 ± 3.3 | 25.2 ± 2.9 ^a,b,c^ | 10.7 ± 3.5 ^a,b,c^ |

**
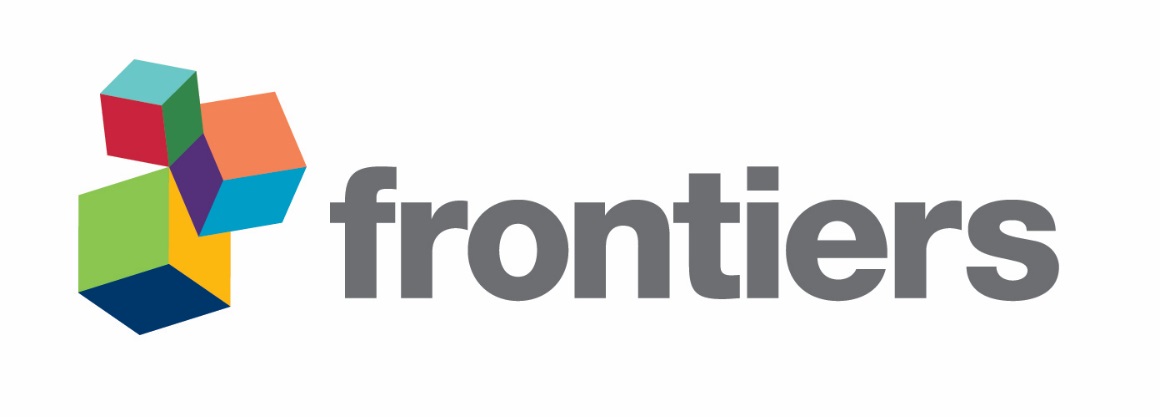
**
